# In vivo inhibition of miR-155 significantly alters post-stroke inflammatory response

**DOI:** 10.1186/s12974-016-0753-x

**Published:** 2016-11-09

**Authors:** Juan Carlos Pena-Philippides, Ernesto Caballero-Garrido, Tamar Lordkipanidze, Tamara Roitbak

**Affiliations:** 1 Department of Neurosurgery, University of New Mexico Health Sciences Center, 1101 Yale Blvd, Albuquerque, NM 87106-3834 USA; 2 Ilia State University, Tbilisi, Georgia

**Keywords:** MicroRNA, miR-155, dMCAO, Post-stroke inflammation, Microglia/macrophages

## Abstract

**Background:**

MicroRNA miR-155 is implicated in modulation of the inflammatory processes in various pathological conditions. In our previous studies, we demonstrated that in vivo inhibition of miR-155 promotes functional recovery after mouse experimental stroke. In the present study, we explored if this beneficial effect is associated with miR-155 inhibition-induced alterations in post-stroke inflammatory response.

**Methods:**

Intravenous injections of a specific miR-155 inhibitor were initiated at 48 h after mouse distal middle cerebral artery occlusion (dMCAO). Temporal changes in the expression of cytokines and key molecules associated with cytokine signaling were assessed at 7, 14, and 21 days after dMCAO, using mouse cytokine gene and protein arrays and Western blot analyses. Electron and immunofluorescence confocal microscopy techniques were used to evaluate the ultrastructural changes, as well as altered expression of specific phenotypic markers, at different time points after dMCAO.

**Results:**

In the inhibitor-injected mice (inhibitor group), there was a significant decrease in CCL12 and CXCL3 cytokine expression at 7 days and significantly increased levels of major cytokines IL-10, IL-4, IL-6, MIP-1α, IL-5, and IL-17 at 14 days after dMCAO. These temporal changes correlated with altered expression of miR-155 target proteins SOCS-1, SHIP-1, and C/EBP-β and phosphorylation levels of cytokine signaling regulator STAT-3. Electron microscopy showed decreased number of phagocytically active peri-vascular microglia/macrophages in the inhibitor samples. Immunofluorescence and Western blot of these samples demonstrated that expression of leukocyte/ macrophage marker CD45 and phagocytosis marker CD68 was reduced at 7 days, and in contrast, significantly increased at 14 days after dMCAO, as compared to controls.

**Conclusions:**

Based on our findings, we propose that in vivo miR-155 inhibition following mouse stroke significantly alters the time course of the expression of major cytokines and inflammation-associated molecules, which could influence inflammation process and tissue repair after experimental cerebral ischemia.

**Electronic supplementary material:**

The online version of this article (doi:10.1186/s12974-016-0753-x) contains supplementary material, which is available to authorized users.

## Background

Ischemic stroke triggers complex, multipart cascade of events leading to irreversible brain damage and dysfunction of the neurovascular network in the ischemic core [1]. Hypoxic but still viable peri-infarct area surrounding the core region remains to be a subject of intensive investigation, with the focus on neuroprotective and pro-regenerative treatments for preservation of salvageable brain tissue. Post-ischemic inflammatory response is an integral part of both brain damage and recovery, yet its function remains controversial. Lower levels of pro-inflammatory cytokines and higher expression of anti-inflammatory cytokines are associated with lower infarct size and a better clinical outcome [2, 3]. On the other hand, number of pro-inflammatory cytokines is implicated in neuroprotection and post-stroke plasticity and, thus, in facilitation of the restorative processes and tissue remodeling [4, 5]. Original elevation of cytokine expression during acute inflammation starts as early as several hours and is associated with recruitment of different types of cells into the ischemic area, including neutrophils, lymphocytes, monocytes, and activation of resident microglia, astrocytes, and endothelial cells [3]. Activation of microglia and astrocytes leading to additional release of pro-inflammatory factors can last for up to several weeks after stroke [5].

Ischemia is associated with significant changes in the molecular profile of the neurovascular elements [6, 7]. Short messenger RNA (mRNA)-interfering molecules microRNAs (miRNAs) are recently identified as essential modulators of inflammatory response in the brain [8, 9]. Multifunctional miRNA miR-155 is among the miRNAs with expression profiles significantly affected by cerebral ischemia [10–12]. miR-155 has been implicated in regulating various physiological and pathological processes including immunity and inflammation [13, 14]. Recent investigations identified miR-155 as a potent regulator of the neuroinflammatory response in Japanese encephalitis and Alzheimer’s disease [15, 16]. Microglia-mediated immune response associated with several pathological states is regulated by miR-155 [17–19]. Specific inhibition of this miRNA is accompanied by reduced inflammation, in several pro-inflammatory conditions [20, 21]. miR-155 mediates the inflammatory response through negative targeting of its direct target proteins including Src homology 2-containing inositol phosphatase 1 (SHIP-1) [15, 22], suppressor of cytokine signaling molecules SOCS-1 and SOCS-6 [19, 23, 24], and transcription factor CCAAT/enhancer binding protein beta (C/EBP-β) [25]. All these miR-155-targeted factors control the inflammatory response via suppression of cytokine transcription and signaling.

In our previous study, we found that specific in vivo inhibition of miR-155 after experimental mouse ischemia supports brain microvasculature in the peri-infarct area, reduces brain tissue damage, and improves the animal functional recovery. In addition, miR-155 inhibition after distal middle cerebral artery occlusion (dMCAO) resulted in alterations of several cytokine/chemokine gene expression [26], prompting our interest to possible role of miR-155 in post-ischemic inflammation. miR-155 is expressed in hematopoietic cells (including B cells, T cells, monocytes, and granulocytes), endothelial cells, microglia, and astrocytes [17, 23, 27]. All these cell types are able to express and secrete pro- and anti-inflammatory molecules and, thus, actively participate in the inflammation process. Therefore, we proposed that systemic in vivo inhibition of miR-155 could significantly affect the post-stroke inflammatory response. While cytokine expression has been mostly studied during the acute inflammation, few studies have examined the temporal profile of pro- and anti-inflammatory molecules during subacute phase of stroke. The present investigation focuses on the long-lasting effect of miR-155 inhibition on the inflammatory response.

To our knowledge, this is a first report describing the effect of intravenous anti-miRNA injections on the time course of cytokine expression and cellular inflammatory response following mouse stroke.

## Methods

### Animal groups

All institutional and national guidelines for the care and use of laboratory animals were followed during the experiments. C57BL/6 male mice (2-month old, Jackson Laboratories; https://www.jax.org/strain/000664) were used in our studies. Experimental groups included sham-operated mice, mice subjected to dMCAO and specific miR-155 inhibitor injections (inhibitor group), and mice subjected to dMCAO and control (scrambled) miRNA inhibitor injections (control group).

### Distal middle cerebral artery procedure

A distal (direct) middle cerebral artery occlusion (dMCAO) was utilized as an experimental model of cortical ischemia [28]. dMCAO was performed on 2-month-old male C57BL/6 mice, as described in [26]. The mice were anesthetized using isoflurane gas (induction dosage 2–3 %; maintenance dosage 1.5–2 %) and a mixture of O_2_:N_2_O gases in the ratio 2:1, delivered during the surgery. The MCA was exposed via transtemporal approach [28, 29]. A small burr hole (located 1 mm rostral to the fusion of zygoma and squamosal bone and 3 mm ventral to the parietal bone) was made on the left side of the skull surface, and the MCA was coagulated with low-heat electrocautery (Bovie Medical). In sham-operated animals, the MCA was exposed but not coagulated.

### miRNA inhibitor injections

Injections of specific anti-miR-155 miRCURY LNA™ (Product#4101082-001, https://www.exiqon.com/microrna-knockdown-probes) inhibitor or control inhibitor (scrambled oligonucleotide, Product#199006) from Exiqon Company were initiated at 48 h after dMCAO and performed for three consecutive days. Oligonucleotides were introduced via mouse lateral tail vein; the dose was 10 mg/kg in saline, total injected volume 100 μl [26].

### Mouse cytokine and chemokine PCR array

Mouse cytokine and chemokine PCR array (Qiagen, Cat# PAMM-150Z, http://www.sabiosciences.com/rt_pcr_product/HTML/PAMM-150A.html) was utilized to evaluate the expression of genes encoding major pro- and anti-inflammatory cytokines and chemokines in the RNA samples. At 7 days after dMCAO, three brains per experimental group (inhibitor and control) were used to generate separate sample triplicates for the analyses. Brain cortices were dissected and stored in RNAlater solution (Ambion). Total RNA was isolated using mirVana miRNA (AB/Ambion) isolation kit, from the hemispheres ipsilateral to dMCAO injury. All measurements and data quantification were performed by Qiagen Company Sample & Assay Technologies team. The obtained raw data were analyzed using RT2 Profiler software (Qiagen). Only the genes with consistent expression levels (within the triplicate samples) were picked up for statistical analysis. The fold changes of gene expressions (inhibitor vs control) were calculated, and the transcripts that showed ≥2-fold change in expression (either up- or downregulated) were retained. At the final step, statistical significance (*p* value <0.05) and reliability of the results was automatically evaluated. The raw data are deposited in the Open Science Framework general data repository, link: https://osf.io/3zhc4/?view_only=0826f6e687884b90ab774328c2746ae1.

### Cytokine protein expression analysis

At 48 h and 7, 14, and 21 days after dMCAO, six brains per experimental group (sham, inhibitor, and control) were used to generate separate samples. Brain cortices from both ipsi- and contralateral (to dMCAO damage) hemispheres were dissected on ice and rapidly frozen. Lesioned and intact hemispheres were analyzed separately. Brain tissue was homogenized in tissue extraction buffer (Life Tech/Invitrogen Cat# FNN0071, 5 ml per 1 g of brain tissue) with the addition of protease inhibitor cocktail (Sigma). The samples were centrifuged at 10,000 rpm for 5 min, and supernatant was collected and kept on ice. Protein concentration was determined for each sample, using DC protein assay kit from BioRad. Brain tissue samples were normalized for total protein content and diluted at 1:10 in assay buffer. Expression levels of CCL12 and CXCL3 were detected using Mouse CCL12/MCP5 PicoKine™ (Boster Biological Technology, Cat# EK1128) and Mouse CXCL3 PicoKine™ ELISA Kits (Boster Biological Technology, Cat# EK1364), according to manufacturer’s recommendations. Other cytokine protein expression was detected using Mouse Cytokine Magnetic 20-Plex Panel Kit (Life Tech/Invitrogen, Cat# LMC0006M, https://www.thermofisher.com/order/catalog/product/LMC0006M), according to the manufacturer’s recommendations. The Panel is designed for the quantitative determination of FGF-basic, GM-CSF, IFN-γ, IL-1α, IL-1β, IL-2, IL-4, IL-5, IL-6, IL-10, IL-12 (p40/p70), IL-13, IL-17, IP-10, KC, MCP-1, MIG, MIP-1α, TNF-α, and VEGF expression. The measurements were done using Luminex xMAP-100 system, at the UNM Center of Molecular Discovery. Cytokine concentrations were calculated automatically, using Specialized Luminex system software. For quantification, only cytokines with consistent expression throughout the samples were retained. Two-way ANOVA followed by Tukey’s multiple comparison test was used for final statistical analysis. The raw data are deposited in the Open Science Framework general data repository, link: https://osf.io/dz5ue/?view_only=4f2c586e7562432595d894b86154b97e.

### Western blot analysis

Five to six brains per experimental group were collected at 7, 14, and 21 days after dMCAO and used to generate separate samples. Brain cortices from ipsi- and contralateral (to dMCAO damage) hemispheres were dissected on ice and rapidly frozen. For tissue lysate preparation, brain tissue was homogenized in tissue extraction buffer (Life Tech/Invitrogen Cat# FNN0071, 5 ml per 1 g of brain tissue) with the addition of protease inhibitor cocktail (Sigma). The samples were centrifuged at 10,000 rpm for 5 min, and supernatant was collected and kept on ice. Protein concentration was determined for each sample, using DC protein assay kit from BioRad. The proteins were separated on 4–20 % gradient Criterion precast gels (Bio-Rad). A broad range molecular weight calibration marker from 10,000 to 250,000 MW (Bio-Rad) was used as a standard. Janus kinase (JAK)/signal transducer and activator of transcription (STAT) signaling pathway analysis was performed using phospho-STAT antibody sampler kit (Cell Signaling Technology Cat#9914, RRID:AB_330385). Other antibodies used were as follows: mouse monoclonal anti-STAT-3 (Cell Signaling Technology Cat# 9139, RRID:AB_331757); rabbit polyclonal anti-SOCS-1 (Cell Signaling Technology Cat# 3950S, RRID:AB_2192983); anti-SHIP-1 (Cell Signaling Technology Cat# 2728, RRID:AB_2126244); anti-C/EBP-β (Cell Signaling Technology Cat# 3087, RRID:AB_2078052); rabbit polyclonal anti-SOCS-6 (Santa Cruz Biotechnology Cat# sc-5608, RRID:AB_661195); rabbit polyclonal anti-Iba-1 (Wako Cat# 019-19741, RRID:AB_839504); rat anti-mouse CD68 (AbD Serotec Cat# MCA1957, RRID:AB_322219); goat polyclonal anti-CD206 (R and D Systems Cat# AF2535, RRID:AB_2063012); and anti-CD45 (R and D Systems Cat# AF114, RRID:AB_442146). Loading was confirmed by comparing actin immunoreactivity across the lanes, using mouse monoclonal anti-actin (Sigma-Aldrich Cat# A2228, RRID:AB_476697). Horseradish peroxidase-labeled secondary antibodies were from Cell Signaling and Amersham Biosciences. The density of the protein bands was determined using ImageJ software (NIH Image, RRID:SCR_003073), normalized by actin expression and quantified using Microsoft Excel software.

### Immunohistochemistry and fluorescence microscopy

The animals were perfused with PBS and 4 % paraformaldehyde (PFA); brains were removed and fixed in PFA and subsequently cryoprotected in 30 % sucrose. The brains were sectioned along the rostral-caudal axis; 16 μm coronal sections were mounted and subjected to immunostaining. Briefly, the slices were post-fixed with 4 % PFA and quenched with 50 mM NH_4_Cl. For antigen retrieval, the mixture of 1 % antigen unmasking solution (Vector Laboratories Cat# H-3301, RRID:AB_2336227) and 10 % of 1 M sodium citrate in PBST (PBS containing 0.05 % Triton X-100) was boiled, and tissue slices were immersed in the mixture for 15 min. Ten percent normal goat serum containing 0.05 % Triton-X was used as a blocking buffer. The slides were subsequently incubated with primary antibodies, at 4 °C overnight. Following antibodies were used for the immunostaining: rabbit polyclonal anti-Iba-1 (Wako Cat# 019-19741, RRID:AB_839504); mouse monoclonal anti-GFAP (BD Biosciences Cat# 556328, RRID:AB_396366); Cy-3-conjugated anti-NeuN (Millipore Cat# ABN78C3, RRID:AB_11204707); goat polyclonal anti-CD206 (R and D Systems Cat# AF2535, RRID:AB_2063012); anti-CD45 (R and D Systems Cat# AF114, RRID:AB_442146); and Alexa Fluor 488-conjugated rat anti-mouse CD68 (AbD Serotec Cat# MCA1957A488, RRID:AB_324822). FITC-, rhodamine-, and Cy-5-conjugated secondary antibodies (1:200 concentration, 2 h at RT) were from Jackson Immunoresearch. DAPI staining was used to visualize nuclei. The incubations were performed in the humidity chamber.

#### Quantification of the immunofluorescence staining intensity

Mouse brain coronal sections co-immunostained for Iba-1/CD206/DAPI or Iba-1/CD45/CD68 were imaged using single-scan and tile-scan imaging on Zeiss LSM510-META and Zeiss LSM-800 Airyscan confocal microscopes. Tile-scan (10 × 15 tiles) imaging performed with ×40 and ×60 magnification allowed us to acquire high-resolution scans of the entire infarct core and peri-infarct areas in the injured hemisphere, as well as to capture a corresponding area in the contralateral hemisphere. Occasionally, Z-stack imaging was used to localize and confirm the intracellular labeling. Three to four coronal sections from the rostral part of the brain (mostly affected by dMCAO) per mouse (*N* = 5–6 per experimental group/per time point) were imaged and quantified. ImageJ software (NIH Image, RRID:SCR_003073) and a modified fluorescence intensity quantification method described in [30, 31] were used for quantification. The brain area of the same size throughout all images (945 × 387 pixels, corresponding to ~500 × 200 μm), including the edge of the infarct (glial scar) and part of the peri-infarct region, was selected and analyzed. The following formula was used for calculations: Corrected total fluorescence of the area = total fluorescence intensity of the selected area − (selected area × mean fluorescence of background readings).

### Electron microscopy

Mice were perfused with 0.5 % glutaraldehyde and 2.5 % paraformaldehyde in 0.1 M Sorensen’s buffer. Cortical tissue (1 × 1 mm) samples were fixed in 2 % osmium, dehydrated, embedded in Araldite, thin-sectioned to 80–90 nm, and stained with 4 % uranyl acetate in methanol followed by lead citrate as described in [26, 32]. Images were acquired using Hitachi H7500 Transmission Electron Microscope equipped with AMT X60 camera. Three to four animals per experimental group (control and inhibitor) were used for analysis; high magnification (×800–15,000) images were taken both in the lesioned and intact hemispheres.

### Statistical analysis

PCR array data were analyzed using RT2 Profiler software (Qiagen). The *p* values were calculated based on a Student’s *t* test of the replicate 2^(−Delta Ct) values for each gene in the control and inhibitor groups. Statistical analysis for all other obtained data was performed using Prism and GraphPad Prism v.6.05, or R software. Two-way ANOVA followed by Tukey’s multiple comparison test was used for cytokine protein expression analysis. Student’s *t* test was performed for the Western blot, immunofluorescence microscopy, and electron microscopy data analyses.

## Results

In our previous study, we characterized miR-155 inhibition efficiency following intravenous injections after mouse dMCAO. Specific anti-miR-155 miRCURY™ LNA inhibitor (Exiqon) probes crossed BBB and were identified both in the blood vessels and brain tissue. RT-PCR detected ~50 % inhibition of miR-155 expression, which lasted for 7 days after the last injection. The functional efficiency of the in vivo miR-155 inhibition was verified by profiling miR-155 target gene and protein expressions [26]. The same injection procedures were performed in the present study: one group of the dMCAO animals received intravenous injections of specific miR-155 inhibitor (inhibitor group), another group—control scrambled oligonucleotide (control group), at 48 h after dMCAO. Injections lasted for three consecutive days. This period of intervention was chosen based on the time course of post-stroke revascularization and inflammation processes. Since miR-155 inhibition is known to have an anti-inflammatory action, we aimed to avoid influencing the acute phase of post-stroke inflammation as an initiator of constructive events, such as activation of microglia and astrocytes, vascular remodeling, and activation of endogenous stem cells. In addition, the intravenous injections would be more effective after the partial restoration of BBB during a subacute phase following dMCAO.

### miR-155 inhibition after dMCAO has a long-term effect on cytokine expression profile

**Fig. 1:**
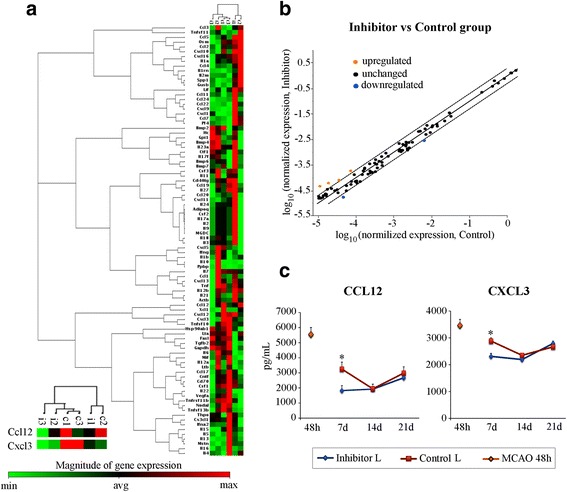
Cytokine expression changes at 7 days after dMCAO. **a** Cytokine gene profiling at 7 days after dMCAO, inhibitor versus control groups. Clustergram of 84 common cytokines grouped by sample type (i1, i2, i3—samples collected from three different mice from the inhibitor group. c1, c2, c3—three samples from control group). Non-supervised hierarchical clustering was used to display common cytokine gene expression by heat map visualization, with dendrograms indicating coregulated genes across groups or individual samples. Results for two statistically different (between inhibitor and controls) genes Ccl12 and Cxcl3 are enlarged and showed in the *bottom left corner*. A *color bar* (*bottom*) relates color code to the magnitude of the differences in gene expression relative to the all-sample means for each gene. The results are also demonstrated in the form of scatter plots (**b**) of 84 common cytokine assays, revealing four upregulated and two downregulated genes (with more than twofold difference in expression between the inhibitor and control groups). *Orange symbols* identify upregulated genes, and *blue symbols* identify downregulated genes. **c** Protein expression levels of CCL12 and CXCL3 cytokines were assessed in the lesioned hemispheres of the animals from control (*red plots*) and inhibitor (*blue plots*) groups at 7,14, and 21 days after dMCAO, using specialized Mouse CCL12 and CXCL3 PicoKine™ ELISA kits (Boster Biological Technology). Cytokine expressions were also measured in the lesioned hemisphere of dMCAO-subjected mice at 48 h post-dMCAO (*orange diamond*). *N* = 6 mice per group/per time point. Two-way ANOVA followed by Tukey’s multiple comparison test; *p < 0.05. *Error bars*: SEM

Initially, we investigated the effect of miR-155 inhibition on the expression of genes encoding pro- and anti-inflammatory factors, at 7 days following dMCAO. Specialized cytokine/chemokine PCR profiler array was utilized for this study. In accordance with already reported data [26], gene profiling revealed a significant (>2-fold; *p* < 0.05) downregulation of two cytokine genes, including pro-inflammatory chemokine ligand 12 (*Ccl12*) and chemokine ligand 3 (*Cxcl3*) (Fig. 1a, and blue symbols on Fig. 1b). Among the upregulated genes, increased expression of genes encoding bone morphogenetic protein 4 (*Bmp4*), cardiotrophin 1 (*Ctf1*), interleukin-23 subunit alpha (*Il-23a*), and interleukin-17 (*Il-17*) were statistically significant but less than twofold different from the control group samples. Colony stimulating factor-3 gene (*Csf3*), chemokine (C-X-C motif) ligand-9 gene (*Cxcl9*), and pro-platelet basic protein gene (*Ppbp*) were increased more than twofold, and interleukin-10 gene (*Il-10*) was upregulated fourfold (Fig. 1a and orange symbols on Fig. 1b), but these elevations were not consistent throughout all samples. mRNA levels of other cytokines in the array were not different between control and inhibitor groups (Fig. 1a, b). We then assessed protein expression of CCL12 and CXCL3 cytokines in the control and inhibitor groups (six mice per group), using specialized quantitative ELISA detection kits. The measurements were performed at 48 h (before the inhibitor injections) and 7, 14, and 21 days after dMCAO. CCL12 concentration at 48 h post-dMCAO was ~5550 pg/mL and decreased in both groups at 7, 14, and 21 days. Protein measurements supported our gene expression data, showing that at 7 days after dMCAO, CCL12 had significantly (1.8-fold) lower expression in the samples from the inhibitor group, as compared to controls (Fig. 1c, left panel). Similarly, at 7 days after stroke, CXCL3 protein levels were lower in the inhibitor group, as compared to controls; this decrease was consistent throughout all samples and statistically significant (Fig. 1c, right panel). At 14 and 21 days, the cytokine concentrations were similar between the groups (Fig. 1c).

**Fig. 2:**
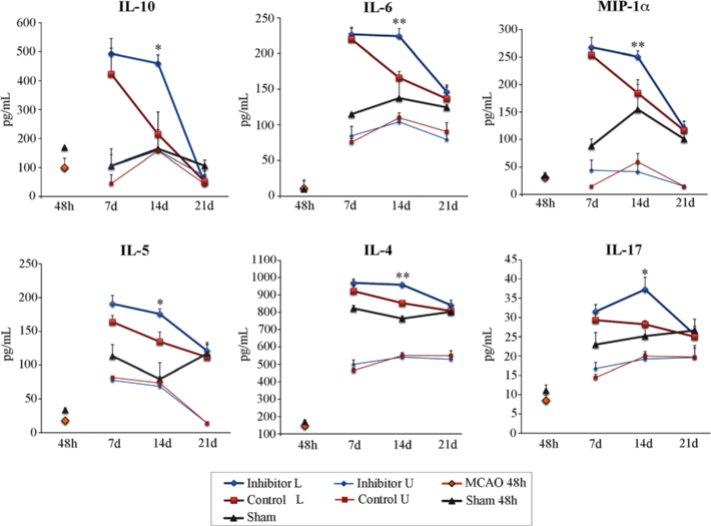
miR-155 inhibition results in long-term changes in cytokine profiles. Protein expression of 20 major pro- and anti-inflammatory cytokines was measured using Mouse Cytokine Magnetic 20-Plex Panel Kit (Life Tech/Invitrogen), at 48 h and 7, 14, and 21 days after dMCAO. Samples were collected from lesioned (*thick red* and *blue plots*) and unlesioned hemispheres (*light red* and *blue plots*) of mice from control and inhibitor groups, respectively. Sham group (*dark grey plots*) did not receive any injections. dMCAO and sham-operated animals were used to measure cytokine expressions at 48 h after dMCAO (*orange diamond* and *grey triangle*). *N* = 6 mice per group/per time point. Two-way ANOVA followed by Tukey’s multiple comparison test; *p < 0.05, **p < 0.01. *Error bars*: SEM

Protein expression of other major pro- and anti-inflammatory cytokines was measured using Mouse Cytokine Magnetic 20-Plex Panel Kit (Life Tech/Invitrogen), at 48 h and 7, 14, and 21 days after dMCAO. The measurements of 20 different cytokine expressions were performed in the tissue extracts collected from lesioned (L, ipsilateral to dMCAO injury) and unlesioned (U, contralateral to dMCAO injury) hemispheres of mice from control and inhibitor groups (six mice per group/per time point). Sham group (three animals per time point) did not receive any injections. In order to measure the cytokine profiles before the initiation of miR inhibition, six dMCAO and six sham-operated animals were sacrificed at 48 h after dMCAO. The major differences between control and inhibitor groups were detected at 14 days after dMCAO. At this time point, the expression of six cytokines was consistently higher in the lesioned hemispheres of the mice from the inhibitor group (thick blue line plots), as compared to controls (thick red plots, Fig. 2). Among significantly upregulated proteins were anti-inflammatory interleukins IL-10 and Il-4 [33, 34]; cytokines with context-dependent dual (both anti- and pro-inflammatory) action, including interleukins Il-6, IL-5, and Il-17 [35–37]; and pro-inflammatory macrophage inflammatory protein-1 family member MIP-1α [38]. At 48 h post-dMCAO, cytokine concentrations in the lesioned hemisphere of dMCAO animals were not significantly different from the sham group (Fig. 2, orange diamonds and grey triangles, respectively). This was expected since, according to the existing literature, cytokine levels initially peak at several hours after ischemia and, in most cases, return to the basic levels by 24–48 h [39]. Cytokine expression in the ipsilateral hemispheres of the inhibitor and control groups significantly increased at 7 days, followed by a notable decrease (almost to the basic levels) at 21 days after dMCAO (Fig. 2). Accordingly, in the inhibitor samples, IL-10 increased fivefold at 7 days after stroke, as compared to expression at 48 h. Delayed (6–7 days) elevation of IL-10, IL-4, and IL-17 in the post-acute phase of stroke was previously reported by other authors [40, 41]. In control group, cytokine concentrations peaked at 7 days after dMCAO and steadily declined at 14 and 21 days (Fig. 2, thick red plots). In contrast, in the inhibitor group, the concentration of all these cytokines retained peak expression at both 7 and 14 days after dMCAO (Fig. 2, thick blue line plots). Cytokine concentrations in the sham group (dark grey plots) also increased at 7 days (with the exception of IL-10) but, until 21 days, remained significantly lower than in the lesioned hemispheres of both inhibitor and control groups. At 21 days, cytokine expression in the lesioned hemispheres was similar between dMCAO and sham animals. IL-10 expression returned to the basic levels, while other cytokines remained slightly elevated at this time point. Cytokine expression in the unlesioned hemispheres of both inhibitor and control groups (light blue and red plots, respectively) remained significantly lower than in the lesioned hemispheres. To summarize, the expression of IL-10, IL-6, MIP-1α, IL-5, IL-4, and IL-17 significantly increased in the lesioned hemisphere at 7 days after dMCAO. In contrast to control animals, in the inhibitor group, high concentration of all six cytokines remained for up to 14 days after stroke. At this time point, the levels of these cytokines were significantly higher in the inhibitor group, as compared to controls.

### miR-155 inhibition results in the impaired cytokine signaling

**Fig. 3:**
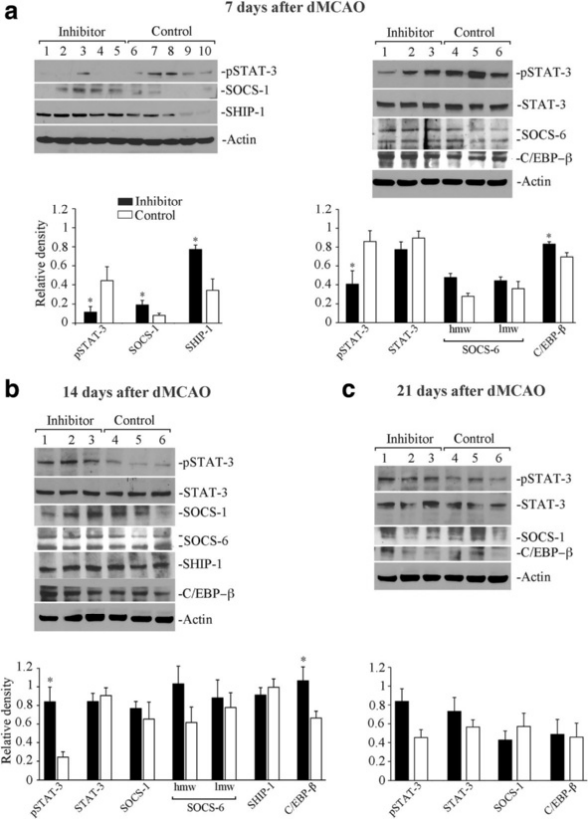
miR-155 inhibition affects cytokine signaling machinery. **a** *Left panel*: Western blot analysis of phospho-STAT-3, SOCS-1, and SHIP-1 expression in lysates prepared from the cortical tissue of the animals from inhibitor and control groups, at 7 days after dMCAO. *Graphs* represent WB quantification analysis; Optical density of each band was normalized to β-actin (loading control). The samples were collected from the lesioned hemispheres of five different mice per group. *Right panel*: higher quantity of the samples described in **a** was loaded in order to detect and quantify phospho-STAT-3, STAT-3, SOCS-6 (with two corresponding 62 kDa (lmw) and 82 (hmw) bands), and C/EBP-β proteins. *Graphs* represent quantification of the relative density representing protein expression normalized to actin expression (*N* = 5–6 animals per group). **b**, **c** p-STAT, STAT-3, SOCS-1, SOCS-6, SHIP-1, and C/EBP-β protein expressions were detected and quantified in the samples collected from the lesioned hemispheres of the inhibitor and control animals at 14 (**b**) and 21 (**c**) days after dMCAO. *N* = 5–6 animals per group. At 21 days, the expression levels of SOCS-6 and SHIP-1 were undetectable (not shown). For all graphs: *error bars*: SEM; Student’s *t* test, *p < 0.05

In order to examine possible molecular mechanisms underlying the effect of miR-155 inhibition on cytokine profiles, we evaluated the expression of key cytokine signaling regulator proteins, in the lesioned hemispheres of the animals from the inhibitor and control groups, at 7, 14, and 21 days after dMCAO. The activity of JAK/STAT signaling cascade, a key regulator of cytokine signaling, was evaluated using phospho-STAT antibody sampler kit (Cell Signaling). In addition, we examined the expression of direct miR-155 targets, including cytokine signaling suppressors SOCS-1, SOCS-6, SHIP-1, and C/EBP-β. At 7 days after dMCAO, expression of phospho-STAT-3 (phosphorylated at Tyr705) was consistently and significantly decreased in the samples from the inhibitor group, indicating decreased activity of STAT-3 and, thus, STAT-3-regulated cytokine signaling (Fig. 3a, left panel and graph). Reduced phosphorylation (activity) of STAT-3 was accompanied by the elevated SHIP-1 and SOCS-1 (negative regulator of STAT-3) in these samples (Fig. 3a, left panel and graph). Since the expression of SOCS proteins is very low in the adult brain, a higher protein loading was required for their analysis. Right panel of Fig. 3a demonstrates that the expression of another STAT regulator SOCS-6 was not substantially affected by miR-155 inhibition, although high molecular weight (82 kDa) isoform of this protein was increased ~1.7-fold in the inhibitor samples, as compared to controls. miR-155 target protein C/EBP-β was significantly increased in the samples collected from the inhibitor-injected animals. While STAT-3 phosphorylation was reduced in the inhibitor samples, STAT-3 protein levels were similar between the groups (Fig. 3a, right panel and graph). We conclude that at 7 days after dMCAO, miR-155 inhibition may suppress cytokine signaling via its direct targets SOCS-1 and SHIP-1. At 14 days following dMCAO, there was a reversal in the STAT-3 activation, with now increased phospho-STAT-3 levels in the inhibitor group (Fig. 3b). Importantly, changes in STAT-3 phosphorylation occurred on Tyr705 residue, which is exclusively affected by JAK-associated cytokine-stimulated receptor signaling [42]. Phosphorylation of other STAT proteins (including STAT-1, STAT-2, STAT-5, and STAT-6) was not clearly detectable in our samples. C/EBP-β remained upregulated in the inhibitor samples. At the same time, the levels of SOCS-1 and SOCS-6, as well as SHIP-1, were similar between the inhibitor and control samples (Fig. 3b). This is in agreement with our findings that in vivo miR-155 inhibition lasts for 10 days after the inhibitor injections, meaning that the microRNA levels were restored by 14 days after dMCAO. Increased phosphorylation of STAT-3 in the inhibitors at this time point can be explained by reduced suppression from SOCS proteins, as well as by increased signaling of STAT-3 activator interleukin IL-10. In turn, IL-10 increase may be induced by the elevated C/EBP-β. At 21 days after dMCAO, no significant differences were detected in STAT-3 phosphorylation, or in the expression levels of SOCS-1 and C/EBP-β, between inhibitor and control groups (Fig. 3c). Expressions of SOCS-6 and SHIP-1 in these samples were undetectable. In conclusion, at 7 days after dMCAO, miR-155 inhibition resulted in de-activation of STAT-3, which signifies suppression of STAT-3-mediated cytokine signaling. In contrast, at 14 days after dMCAO, there was an activation of STAT-3 in the miR-155 inhibitor-injected animals. Prolonged elevation of anti-inflammatory IL-10 (possibly facilitated by still upregulated C/EBP-β) and pSTAT-3 could trigger IL-10/STAT-3-mediated anti-inflammatory response. No differences between the animal groups were detected at 21 days after dMCAO, which correlates with our cytokine expression analysis.

### Temporal distribution of microglia and astrocytes after dMCAO

**Fig. 4:**
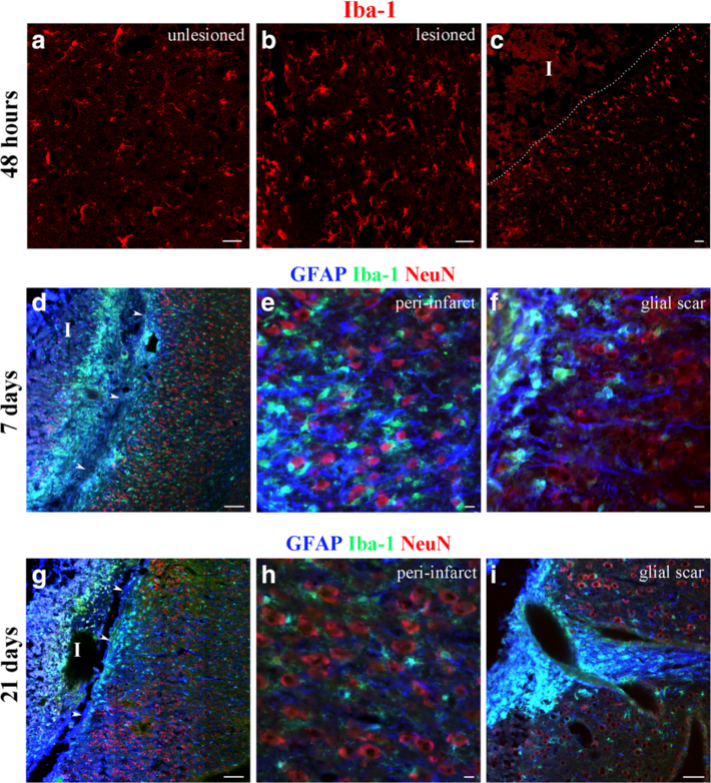
Fluorescence microscopy analysis of the cellular inflammatory response during the subacute phase of stroke. Coronal sections of the unlesioned (**a**) and lesioned (**b**, **c**) hemispheres of mice subjected to dMCAO, at 48 h after surgery. The sections were immunostained with anti-Iba-1 antibody (*red*). **c** Peri-infarct area of stroke; Infarct core (I) is outlined with *white dotted line*. Note the transformation of microglial phenotype from ramified to hypertrophic bushy and ameboid, as their position is closer to the infarct border. **d**–**f** 7 days after dMCAO. Triple immunostaining with the antibodies against GFAP (*blue*), Iba-1 (*green*), and NeuN (*red*). **d** Infarct core (I), glial scar, and peri-infarct area. The edge of the infarct is marked with *white arrowheads*. **e**, **f** Higher magnification images demonstrate the distribution of microglia (*green*), astrocytes (*blue*), and neurons (*red*) in the peri-infarct area and the immediate vicinity to glial scar. **g**–**i** 21 days after dMCAO. **g** The infarct core (I), glial scar, and peri-infarct area. The edge of the infarct is marked with *white arrowheads*. Higher magnification images demonstrate the distribution of microglia (*green*), astrocytes (*blue*), and neurons (*red*) in the peri-infarct area (**h**). Note the narrowing of the glial scar and its concentration mainly in the white matter area (**i**). Imaging was performed using Zeiss LSM510-META confocal microscope, using single-scan and tile-scan image acquisitions. *Bars*: **a**–**c** 20 μm; **d**, **g** 100 μm; **e**, **h** 10 μm; **f**, **i** 50 μm

Immunofluorescence microscopy was used to assess the distribution and phenotype of the cells responsible for post-stroke inflammatory response, including microglia/macrophages and astrocytes (the term microglia/macrophages will be substituted with microglia/macrophages (M/Ms), throughout the text). At 48 h following dMCAO, activated microglia were seen throughout whole ipsilateral (lesioned) hemisphere (Fig. 4b). Hypertrophic cells with a strong expression of microglia marker Iba-1 were substantially different from the resting ramified microglia in the contralateral hemisphere (Fig. 4a). In the peri-infarct area, hypertrophied microglia exhibited bushy morphology, which gradually transformed into mostly ameboid phenotype in the vicinity of the infarct edge (Fig. 4c). At 7, 14, and 21 days, hypertrophied activated microglia were mostly restricted to the peri-infarct zone. Glial scar was well established by 7 days following dMCAO and consisted of ameboid microglia (predominantly in the inner border zone) and more peripherally localized reactive astrocytes (Fig. 4d–f). There was a significant infiltration of Iba-1-positive M/Ms into the infracted tissue (Fig. 4d; I—infarct core, infarct edges are marked with arrowheads). Based on the literature, these invading cells mostly consist of the macrophages, which are recruited and activated by the hypoxic/ischemic environment of the infarct core [43]. At 21 days post-MCAO, glial scar was narrowed down, and both M/Ms and astrocytes of the peri-infarct zone exhibited lower degree of reactivity, as compared with 7 days after stroke (Fig. 4g, h). Most of the activated M/Ms and astrocytes remained at the edge of infarct, in the area of the white matter (Fig. 4i). The distribution of microglia and astrocytes was similar between the inhibitor and control groups, at all examined time points after dMCAO.

### Ultrastructural changes associated with miR-155 inhibition

**Fig. 5:**
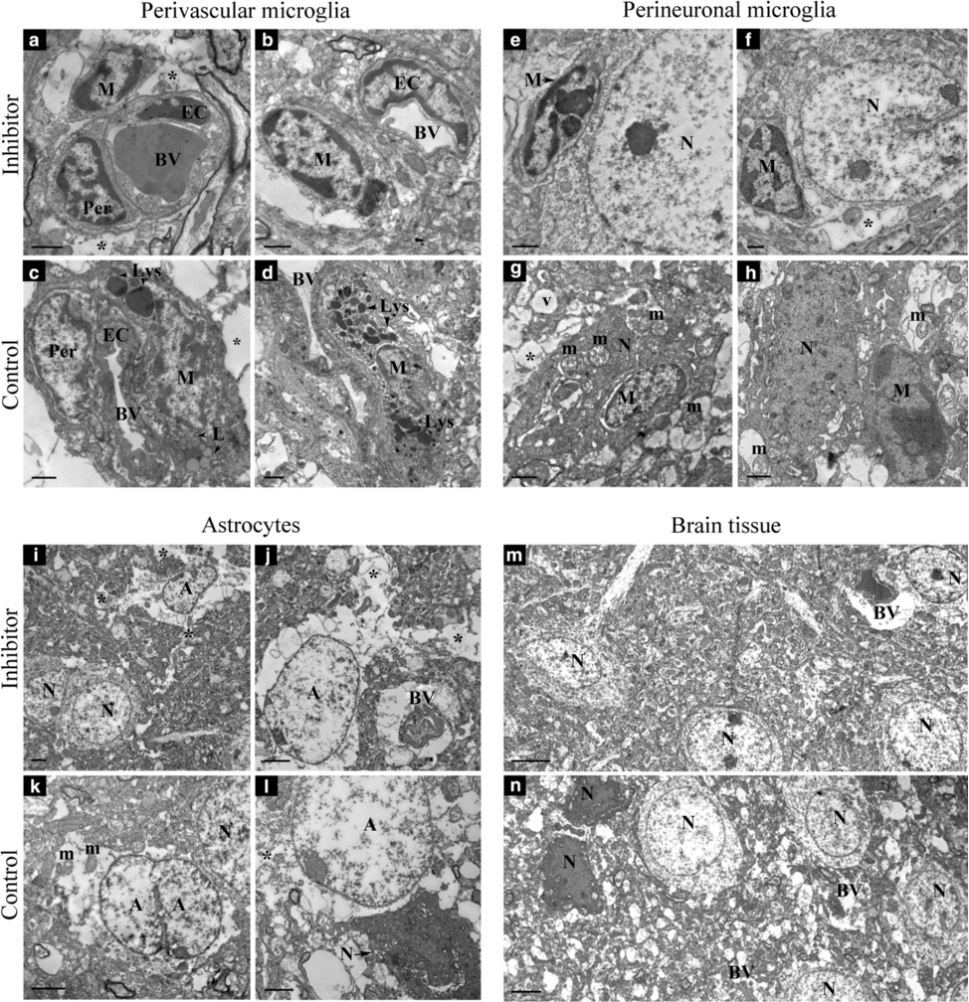
Ultrastructural characteristics of microglia and astrocytes during subacute phase of post-stroke inflammation. Representative transmission electron microscopy images of capillaries, neurons, microglia, and astrocytes in the peri-infarct area of stroke, at 7 and 21 days after dMCAO, in the inhibitor and control animals. **a**–**d** At 7 days post-dMCAO, perivascular M/Ms were seen in the vicinity of the microvessels. Note the signs of significant phagocytic activity of the perivascular microglia in the control group, with characteristic large lysosomal inclusions in the cytoplasm (**c**, **d**). **e**–**h** Perineuronal microglial cells were seen adhered to the healthy-looking neurons in the inhibitor group (**e**, **f**) and damaged necrotic neurons in the control group (**g**, **h**), at 7 and 21 days after dMCAO. Microglia nuclei contained distinctly accumulated dense heterochromatin. **g** Microglial cell invaded the damaged neuron. Neuronal cytoplasm has dispersed and disrupted organells and swollen mitochondria. **i**–**l** Swollen activated perineuronal and perivascular astrocytes were seen in both groups, at 7 and 21 days after dMCAO. The astrocytes were characterized by dispersed residual organelles and swollen mitochondria. Astrocyte fusion was often observed (**k**). **m**, **n** Significant edema and neuronal necrosis were observed at 21 days in control animals, with multiple necrotic neurons, collapsed blood vessels, and heavily vacuolated brain tissue (**n**). In contrast, visibly less damaged brain tissue was observed in the animals from the inhibitor group. Note healthy-looking neurons, intact and functional microvessels, and overall normal appearance of neuropil (**m**). *M* microglia/macrophage, *BV* blood vessel, *EC* endothelial cell, *Per* pericyte, *Lys* lysosome, *L* lipofuscin-like deposit, *N* neuron, *A* astrocyte, *—astrocytic process, *m*—mitochondria. *Bars*: **a**–**h** 1 μm; **i**–**l** 2 μm, and **m**, **n** 5 μm. Original magnifications: **a**–**l** ×1500–7000 and **m**, **n** ×800

Electron microscopy studies were performed for detailed evaluation of the microglia and astrocyte phenotype in the peri-infarct area of stroke. In previous studies, we reported discrete electron microscopic changes in structural elements of the peri-infarct area of stroke observed for as long as 21 days following dMCAO. These changes included damaged and collapsed microcapillaries with disrupted tight junctions at 7 days, followed by a significant neuronal damage at 21 days after dMCAO. We demonstrated that miR-155 inhibition supported capillary integrity and prevented prolonged neuronal death in the peri-infarct area [26]. The present study was focused on the ultrastructure of the brain tissue elements involved in post-stroke inflammatory response. Perivascular M/Ms were seen in close vicinity of the capillaries (Fig. 5a–d). In the control group, perivascular M/Ms were often increased in size and characterized by intense phagocytic activity. The cytoplasm of these hypertrophied cells contained rich lysosomal apparatus with well-developed lipophagosomes and numerous lipofuscin-like deposits (Fig. 5c, d). In total, 90 capillaries in the control group and 114 capillaries in the inhibitor group were imaged and analyzed. The quantification analysis revealed that 14 % of the examined capillaries in the control group was accompanied by the perivascular M/Ms with high phagocytic activity. In contrast, only 3.5 % of capillaries in the inhibitor group (significantly lower as compared to controls, *p* < 0.05) were associated with the perivascular phagocytic cells. According to the literature, phagocytically active perivascular microglia contribute to disintegration of BBB during the post-stroke inflammation [44]. Therefore, perivascular M/M quantification data are in agreement with our previous findings that at 7 days after stroke, vascular permeability was significantly lower in the inhibitor group, as compared with controls [26]. Activated microglial cells were frequently present in a close vicinity of neuronal soma (Fig. 5e–h). In the control groups, perineuronal microglia tightly adhered to and often invaded the degenerating “dark” neurons (Fig. 5g, h). Electron microscopic alterations in astroglia were similar between the control and inhibitor groups and included significant swelling and degenerative changes in astrocytic perikarya and their processes; these pathological changes were seen in perivascular astrocytes, as well as in the astrocytes adhering to neurons or distributed in the neuropil. The cytoplasm of the remarkably swollen astrocytes was sometimes completely empty, with dispersed residual organelles and swollen mitochondria (Fig. 5i–l). In the control group, swollen astrocytes adhered to damaged and necrotic neurons (Fig. 5l). Micrographs on panels m and n demonstrate the neurons, capillaries, and neuropil in the peri-infarct area of the brains from the inhibitor (m) and control (n) groups, at 21 days after stroke. In agreement with our previous studies, in the inhibitor group, these brain tissue elements have less damaged and more viable appearance. In contrast, in the control group, we observed substantial number of necrotic and damaged neurons, collapsed blood vessels, and significant tissue edema. These observations are in agreement with our previous findings demonstrating 46 % lower amount of damaged neurons, and 21 % lesser collapsed blood vessels in the inhibitor group as compared with controls, at 21 days after dMCAO [26]. Thus, in the inhibitor animals, lower number of phagocytically active perivascular M/Ms correlates with stabilized microvasculature, decreased brain tissue edema, and reduced neuronal damage.

### Effect of miR-155 inhibition on the expression of phenotypic markers

**Fig. 6:**
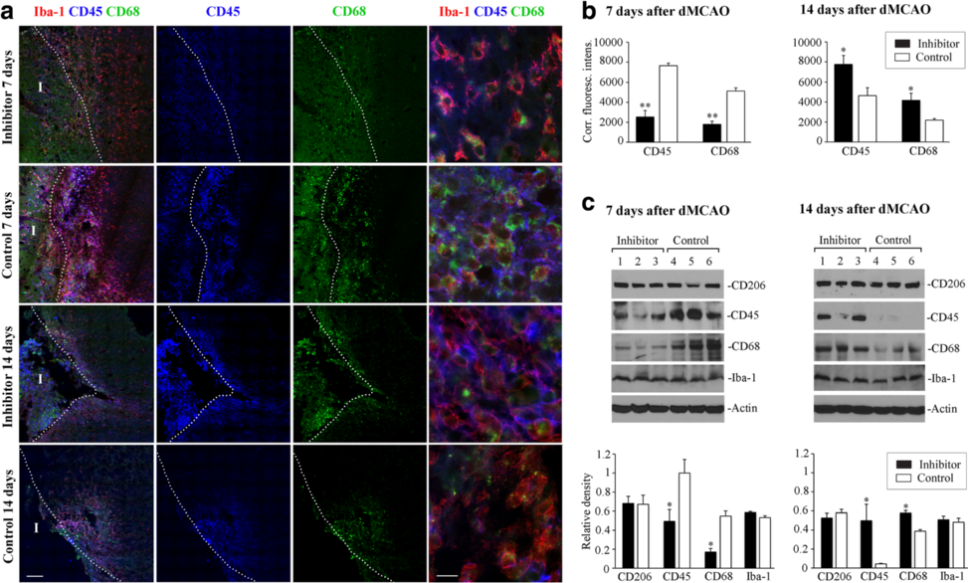
Analysis of M/M phenotypic markers at different time points after dMCAO. **a** Coronal sections of the lesioned hemispheres of the inhibitor and control mice brains, at seven (*two upper rows of panels*) and 14 days (*two lower rows*) after dMCAO. The sections were triple-immunostained with anti-Iba-1 (*red*), anti-CD45 (*blue*), and anti-CD68 (*green*) antibodies. Representative merged and single-channel images depict part of the infarct core (I, outlined with *white dotted lines*) and peri-infarct area of stroke. High magnification images demonstrate distribution of Iba-1, CD45, and CD68 in the glial scar area (*right panels*). Imaging was performed using Zeiss LSM800 Airyscan confocal microscope, using single-scan and tile-scan image acquisitions. *Bars*: from left to right: 100 and 10 μm. **b** Quantification of the corrected fluorescence intensity for CD45 and CD68 immunofluorescence staining in the inhibitor (*black bars*) and control groups (*white bar*), at 7 and 14 days after dMCAO. *N* = 5–6 mice per group/per time point, three brain sections per mouse. *Error bars*: SEM; Student’s *t* test, *p < 0.05; **p < 0.01. **c** Representative Western blots and quantification analysis (*graphs*) of CD206, CD45, CD68, and Iba-1 expression in lysates prepared from the lesioned cortical tissue of the animals from the inhibitor (*black bars*) and control (*white bars*) groups, at 7 and 14 days after dMCAO. *Graphs*: optical density of each band was normalized to β-actin (loading control). *N* = 5–6 mice per group/per time point. *Error bars*: SEM; Student’s *t* test, *p < 0.05

miR-155 is known to regulate a differentiation process in microglia and macrophages [19, 45]. We therefore investigated the effect of in vivo miR-155 inhibition on the expression of several M/M phenotypic markers, at different time points after dMCAO. We used immunofluorescence staining and Western blot for these experiments, since brain tissue disintegration (both without and with fixation) affects detectability of cell surface markers and, thus, accuracy of FACS-based analysis [46]. Immunofluorescence staining of the histological sections did not show visible differences in CD206 (C-type lectin mainly present on the surface of macrophages and immature dendritic cells) expression between inhibitor and control groups, at any examined time point after dMCAO (Additional file 1). These observations were later supported by Western blot analysis (Fig. 6c). CD206 was expressed in hypertrophied Iba-1-positive cells, where it displayed both membrane and intracellular localization. These Iba1/CD206 expressing cells were mostly localized to the glial scar region, at all examined time points after dMCAO (Additional file 1). Similar distribution of CD206 after stroke was previously reported by other authors [47]. We then performed immunofluorescence staining for other phenotypic markers including CD45 antigen (tyrosine phosphatase, type C, expressed in the majority of hematopoietic cells, including leukocytes and macrophages) and marker for active phagocytosis CD68 (glycoprotein that is highly expressed by monocytes and tissue macrophages). Triple immunostaining for Iba-1, CD45, and CD68 revealed visible differences in the intensity of the expression of CD45 and CD68, between inhibitors and controls, at 7 and 14 days after dMCAO. CD45- and CD68-positive cells were exclusively localized to the glial scar area; some of them infiltrated the infarct core region (Fig. 6a, left column of panels). At 7 days after dMCAO, we observed distinctly lower intensity of CD45 and CD68 expression in the inhibitor group, as compared to controls (Fig. 6a, two upper rows). Quantification of the fluorescence intensity (Fig. 6b) and Western blot analysis (Fig. 6c) confirmed this observation. In contrast to 7 days, at 14 days after dMCAO, intensity of both CD45 and CD68 was higher in the inhibitor group, as compared to controls (Fig. 6a–c). No differences between groups were observed at 21 days after dMCAO (not shown). Western blot analysis confirmed that CD206 and Iba-1 expressions were not significantly different between the inhibitor and control groups, at any time point after dMCAO (Fig. 6c). High-resolution immunofluorescence confocal microscopy also showed that CD45, as a cell membrane protein, had a primarily cell surface localization, while CD68, as a lysosomal/endosomal-associated membrane glycoprotein, had mostly intracellular and weak cell membrane expression. In addition, Iba-1, CD45, and CD68 markers were often expressed with different intensity in different cells (Fig. 6a, right column of micrographs). This reflects diversity in the expression of phenotypic markers in the injured brain and the existence of multiple intermediate states of M/M phenotype. Analysis of the phenotypic marker expression at different time points after dMCAO demonstrates that miR-155 inhibition induces temporal shift in the expression of CD45 and CD68 markers. Reduced CD45/CD68 at 7 days corresponds to our EM data showing lower number of phagocytic cells in the inhibitor group. Most importantly, temporal changes in CD45 and CD68 levels in the inhibitor group correlate with the time course of cytokine suppression and activation at 7 and 14 days, respectively.

## Discussion

In our previous study on the in vivo inhibition of miR-155 after mouse dMCAO, we detected that the vascular support achieved by anti-miR-155 injections at 7 days after the experimental stroke played a critical role in the prevention of delayed neuronal loss in the peri-infarct area at the later stages. Our previous analysis showed significant (34 %) reduction of infarct size in miR-155 inhibitor-injected animals at 21 days after dMCAO. Reduced brain injury and preservation of brain tissue reflected in efficient functional recovery of inhibitor-injected animals: bilateral asymmetry/adhesive removal and gait/locomotion tests demonstrated that the mice from the inhibitor group regained their sensorimotor deficits faster than controls [26]. Based on these data, we assumed that vascular support achieved by miR-155 inhibition played a critical role in the prevention of neuronal loss in the peri-infarct area and, thus, in the improvement of functional outcome after stroke. We proposed that increased blood flow and improved vascular integrity could be mainly attributed to miR-155 inhibition-induced stabilization of tight junction (TJ) protein ZO1, via the upregulation of miR-155 target protein Rheb. Here, we hypothesized that, in addition to this possible mechanism, downregulation of pro-inflammatory miR-155 could improve stroke outcome by significantly influencing a post-stroke inflammation. At 7 days after dMCAO, there was a decreased mRNA and protein expression of pro-inflammatory cytokines CCL12 (also known as monocyte chemotactic protein MCP-5) and CXCL3 in the inhibitor group. Both cytokines are known to trigger vascular inflammation via activation of monocyte adhesion and migration through the vascular endothelium, as well as atherosclerotic plaque formation [48, 49]. Seven days time point was also accompanied by significant upregulation of miR-155-targeted proteins. miR-155 direct targets SOCS-1, SOCS-6, and SHIP-1 inhibit cytokine signaling, using multiple suppression mechanisms [50, 51]. Another miR-155 target C/EBP-β promotes the expression of anti-inflammatory cytokines (including IL-10) and contributes to injury repair and neuroprotection [52, 53]. In the inhibitor samples from 7 days after dMCAO, upregulation of SOCS-1 was accompanied by reduced STAT-3 phosphorylation. Cytokine signaling is mediated via essential JAK/STAT pathway, in which STAT phosphorylation is an indicator of cytokine signaling activation [54]. SHIP-1 upregulation corresponds with dephosphorylation of its direct target protein Akt, demonstrated earlier [26]. Together, these data indicate that at 7 days post-dMCAO, miR-155 inhibition potentially suppresses STAT-3- and PI-3K-mediated cytokine signaling via its direct targets SOCS-1 and SHIP-1 activity. We speculate that miR-155 inhibition-associated upregulation of SOCS-1 and SHIP-1 could suppress number of different cytokines, without affecting their mRNA or protein levels. Altered cytokine expression and signaling (as compared to control group) at 7 days after dMCAO was also accompanied by alterations in perivascular M/M phenotype. Electron microscopy imaging revealed that, in contrast to inhibitors, peri-vascular microglia in the control group was characterized by high phagocytic activity. Engulfment of blood vessels by phagocytic microglia results in the degradation of blood vessels and the active breakdown of the BBB. It is suggested that phagocytic microglia actively participate in the transfer of stroke-induced injury in healthy neighboring tissue by the disassembly of blood vessels and the resulting decrease in blood flow in the ischemic penumbra [44]. Thus, present EM data are in good correlation with our previous findings that at 7 days post-dMCAO, 30 % in control animals had disrupted tight junctions, as opposed to only 9 % in the inhibitor group. In addition, at the same time point after dMCAO, cerebral blood flow in the peri-infarct area of the inhibitor animals was significantly higher than in controls [26]. Immunofluorescence analysis of the brain sections from 7 days after dMCAO also confirmed decreased expression of leukocyte/ macrophage marker CD45 and active phagocytosis marker CD68 in the anti-miR-155-injected group. Taken together all these findings, we concluded that miR-155-induced suppressed cytokine signaling at 7 days, accompanied by decreased M/M phagocytic activity, could contribute to preservation of TJs observed at 7 days after dMCAO.

In the inhibitor group, expression of several important cytokines lasted for as long as 14 days after dMCAO (in contrast to a sharp decline after 7 days, detected in the control samples). Sustained increase in expression of IL-10 in the inhibitor-injected animals is in agreement with recent investigations demonstrating suppression of this cytokine by miR-155 [55]. This major anti-inflammatory cytokine was shown to trigger anti-inflammatory response beneficial for stroke outcome [41, 56]. Significantly higher (as compared to controls) expression of five other cytokines was also detected in the inhibitor group at 14 days after dMCAO. These cytokines exhibiting context-dependent dual action are implicated in having a significant impact on neuroprotection and overall stroke outcome. Il-4 (mostly regarded as anti-inflammatory cytokine) and IL-5 were found to play a beneficial role in brain repair, modulate microglial response, and suppress post-stroke inflammation [57–59]. IL-6, apart from its well-known pro-inflammatory function, can also exhibit neurotrophic and regenerative features following cerebral ischemia [60, 61]. MIP-1α, a member of the CC chemokine subfamily, is upregulated at early acute stages of cerebral ischemia and may have a role in promoting inflammatory and/or repair processes in ischemic brain [62]; at later stages, this chemokine can also serve as a chemoattractant for stem cell migration after ischemic injury [63]. The late (6 days after stroke) peak of IL-17 was detected in other studies, proposing a possible role of this cytokine in neovascularization after MCAO [40]. High levels of IL-10 at 14 days after dMCAO correlated with and could be induced by sustained elevation of C/EBP-β detected at 7 and 14 days after dMCAO. In addition, increased IL-10 levels at 14 days could be associated with the reduced suppression from SOCS-1 and SHIP-1 [55]. STAT-3 activation at this time point could be associated with (a) reduced suppression from SOCS-1 and (b) sustained upregulation of IL-10. IL-10/STAT-3 pathway activation triggers IL-10-mediated anti-inflammatory response (AIR): upon IL-10-induced activation, STAT-3 stimulates the expression of AIR factors, which specifically suppress pro-inflammatory cytokine signaling [33, 64]. Based on the literature and our previous studies, miR-155 inhibition could potentiate IL-10/STAT-3-mediated AIR, which could significantly contribute to improved post-stroke recovery reported in our previous study [26].

Since miR-155 is implicated in the regulation of macrophage differentiation and polarization toward anti-inflammatory phenotype, we expected that miR-155 inhibition would lead to an increased expression of the anti-inflammatory phenotype marker CD206. Instead, we detected altered expression of CD45 and CD68 markers. Increased CD45 expression in the inhibitor group (as compared to controls) at 14 days after stroke may be associated with prolonged upregulation of IL-10, since IL-10 was found to activate CD45 protein tyrosine phosphatase [65]. CD45 is regarded as a negative regulator of pro-inflammatory microglia activation associated with MAPK signaling propagation and neuronal death [66]. According to the literature, Iba-1/CD45-positive macrophages expressing active phagocytosis marker CD68 facilitate brain recovery process following LPS-induced brain injury [67] and stroke [68] and mediate neuroprotection in Alzheimer’s disease [69]. The upregulation of CD68 and, thus, phagocytic activity at 14 days after dMCAO could facilitate removal of debris and dead tissue, as well as promote revascularization, neuroprotection, neurogenesis, and overall recovery [70]. Based on the literature, ameboid Iba-1-positive M/Ms populating glial scar area are represented by macrophages, that infiltrated the site of the injury at early post-stroke stages, and resident microglia, that adopted macrophage-like morphology in response to ischemia. Following cerebral ischemia, initially, pro-inflammatory M/Ms undergo differentiation at later stages after stroke and acquire anti-inflammatory pro-regenerative features, as described in [71]. Based on our data, we propose that miR-155 inhibition at 48 h after stroke results in suppression of early transient harmful actions of the activated M/Ms at 7 days, followed by enhancement of their protective and reparative actions at 14 days after dMCAO. In the present study, we did not aim to phenotypically distinguish between the resident microglia and monocyte-derived infiltrated macrophages, or different types of infiltrated leukocytes. This issue will be addressed in our upcoming studies involving Cx3cr1^GFP/+^ /Ccr2^RFP/+^ transgenic mice; this approach will minimize the artifacts associated with cell isolation/sorting and, thus, assure a truly reliable separation of two different cell types comprising the M/M population.

## Conclusions

In summary, we found that in vivo inhibition of miR-155 following mouse experimental stroke significantly affects the time course of the expression of major cytokines and inflammation-associated molecules during a subacute phase after dMCAO. This process could be mediated via the changes in temporal expression of direct miR-155 target proteins. These findings contribute to understanding of the molecular mechanisms underlying a positive effect of miR-155 inhibition on the overall post-stroke recovery reported in our previous studies.

## Additional file

Additional file 1: Fluorescence microscopy analysis of CD206 expression at different time points after dMCAO. Coronal sections of the lesioned hemispheres of the mice subjected to dMCAO, at 48 h and 7, 14, and 21 days after surgery. The sections were immunostained with anti-Iba-1 (red) and anti-CD206 (green) antibodies; DAPI (blue) was used for nuclear staining. Left panels depict part of the infarct core (I) and peri-infarct area of stroke. High magnification images demonstrate distribution of Iba-1 and CD206-positive cells in the peri-infarct area of stroke (middle panels) and glial scar (right panels). Note that CD206 was mostly expressed in the Iba-1-positive ameboid M/Ms populating glial scar area. Imaging was performed using Zeiss LSM510-META confocal microscope, using single-scan and tile-scan image acquisitions. Bars: from left to right: 100, 10, and 10 μm. (TIF 11901 kb)

## Abbreviations

C/EBP-β: Transcription factor CCAAT/enhancer binding protein beta
Ccl12: Chemokine ligand 12
Cxcl3: Chemokine ligand 3
dMCAO: Distal middle cerebral artery occlusion
JAK: Janus kinase
M/M: Microglia/macrophage
MIP-1α: Macrophage inflammatory protein-1 alpha
miRNA: MicroRNA
SHIP-1: Src homology 2-containing inositol phosphatase 1
SOCS: Suppressor of cytokine signaling molecule
STAT: Signal transducer and activator of transcription
