# Supplementary figures and images for: In vivo inhibition of miR-155 significantly alters post-stroke inflammatory response
Source: J Neuroinflammation. 2016 Nov 9;13:287. doi: 10.1186/s12974-016-0753-x (PMC5103429; doi:10.1186/s12974-016-0753-x)

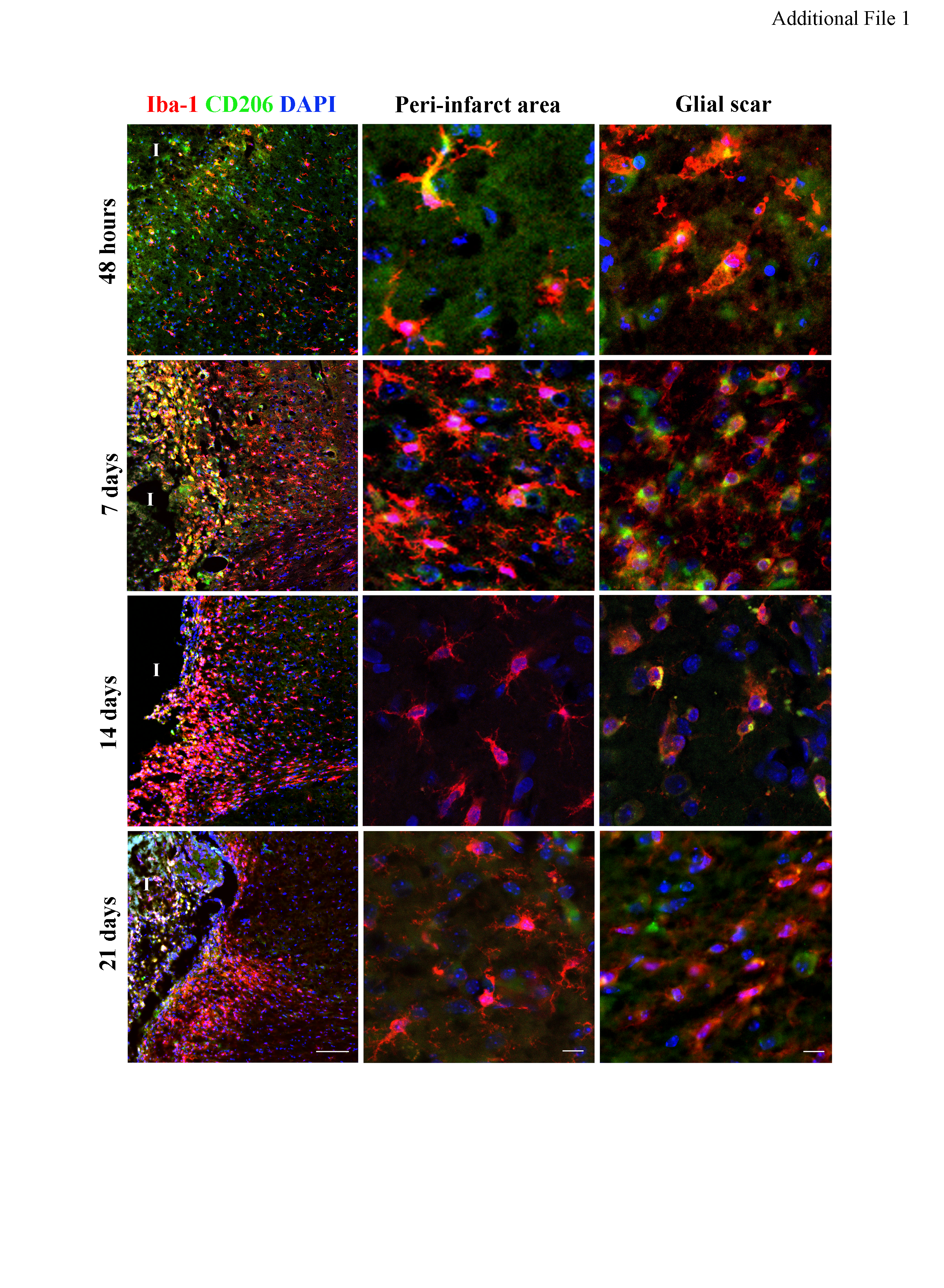

Supplement: Additional file 1: — Fluorescence microscopy analysis of CD206 expression at different time points after dMCAO. Coronal sections of the lesioned hemispheres of the mice subjected to dMCAO, at 48 h and 7, 14, and 21 days after surgery. The sections were immunostained with anti-Iba-1 (red) and anti-CD206 (green) antibodies; DAPI (blue) was used for nuclear staining. Left panels depict part of the infarct core (I) and peri-infarct area of stroke. High magnification images demonstrate distribution of Iba-1 and CD206-positive cells in the peri-infarct area of stroke (middle panels) and glial scar (right panels). Note that CD206 was mostly expressed in the Iba-1-positive ameboid M/Ms populating glial scar area. Imaging was performed using Zeiss LSM510-META confocal microscope, using single-scan and tile-scan image acquisitions. Bars: from left to right: 100, 10, and 10 μm. (TIF 11901 kb) [file 12974_2016_753_MOESM1_ESM.tif]
